# Cigarette smoke modulates methylation levels of LEF1-AS1 and impedes its expression: An experimental study

**DOI:** 10.18332/tid/203507

**Published:** 2025-05-02

**Authors:** Bader O. Almutairi, Ahmed Rady, Bashayer S. Aljuhani, Mikhlid H. Almutairi

**Affiliations:** 1 Department of Zoology, College of Science, King Saud University, Riyadh, Saudi Arabia

**Keywords:** cigarette smoke, DNA methylation, LEF1-AS1, mRNA expression, HUVECs

## Abstract

**INTRODUCTION:**

Cigarette smoke (CS) contains carcinogenic substances and influences genetic regulation and epigenetic modifications, such as DNA methylation. It plays a role in the development of various cancers, including colon, bladder, lung cancer, and leukemia. Long non-coding RNAs play a significant role in controlling several pathways in the cell, including lymphoid enhancer-binding factor 1 antisense RNA 1 (LEF1-AS1), which is found overexpressed in lung, oral, glioblastoma, and colon cancers and downregulated in leukemias. We investigated the impact of CS on DNA methylation of the promoter region of LEF1-AS1 as well as its expression in endothelial cells.

**METHODS:**

This experimental study was designed to investigate the effects of cigarette smoke on the methylation status of the promoter region of LEF1-AS1 in smoker and non-smoker samples and its expression in relevant cell models. To measure the alternations of DNA methylation, extracted DNA samples from 64 male subjects (32 smokers and 32 non-smokers) were bisulfite-treated and amplified using polymerase chain reaction (PCR) with methylation-specific PCR primers. Furthermore, to define the impact of CS on LEF1-AS1 expression, human umbilical vein endothelial cells (HUVECs) were fed with media containing CS for 3 and 6 hours. The expression analysis of LEF1-AS1 was performed using the GTEx (Genotype-Tissue Expression) database, including an assessment of its expression in various cancers such as lung and brain cancers. The functional analysis of the LEF1-AS1 gene was conducted across multiple tissues using data from the GENT2 databases, along with meta-survival and functional enrichment analysis.

**RESULTS:**

The results indicated an average increase of 19.8% in DNA methylation of the promoter region of LEF1-AS1 in the samples from the smokers compared with those from the non-smokers, as well as a significant reduction of LEF1-AS1 expression level in the HUVECs (45% and 83%) after treatment with CS (3 and 6 Hours), respectively. LEF1-AS1 expression varied significantly across tumor types when compared to their normal counterparts. Some cancers, such as lung and brain, showed increased expression, suggesting cancer-specific overexpression of LEF1-AS1. Variability in expression across cancers and normal tissues implies potential heterogeneity in gene regulation. A meta-survival analysis of the LEF1-AS1 gene (e.g. GSE31546, GSE31548, GSE19188), revealed hazard ratios (HR) ranging widely, with some studies (e.g. GSE31546, HR=12.02) suggesting increased risk, though confidence intervals often included 1, indicating uncertainty. Low heterogeneity (I^2^=16%, p=0.26) suggests consistency among studies, but the overall findings lack strong statistical significance.

**CONCLUSIONS:**

Our findings indicate that CS alters LEF1-AS1 DNA methylation and causes an inhibition of LEF1-AS1 expression.

## INTRODUCTION

Cigarette smoke (CS) has a negative influence on most organs of the body, contributing to disease progression, including cardiovascular and lung diseases^1^. Most deaths are attributed to CS, and smoking cessation could potentially increase life expectancy by a decade^2^. CS harbors carcinogenic compounds such as nitrosamines and polycyclic aromatic hydrocarbons, which presumably cause dysregulation in gene expressions via the alteration of either single nucleotide polymorphism (SNP) or epigenetic, leading to cancer progression^3^, for example: lung cancer^1^, breast cancer, colon cancer, and acute myeloid leukemia (AML)^4^. In addition, CS enhances the formation of secondary cancers^5^. Studies have also demonstrated an association between smoking parents and an incidence of childhood AML^4^. Besides altering gene expressions, exposure to CS could also influence epigenetic alternations, such as DNA methylation, which silences tumor suppressor genes and enhances the expression of oncogenes^6^. DNA methylation is a key epigenetic modification that involves adding a methyl group (-CH_3_) to the 5-carbon position of cytosine residues within CpG dinucleotides, predominantly occurring in CpG islands found in promoter regions. This modification is critical in regulating gene expression by influencing chromatin structure, transcription factor binding, and RNA polymerase recruitment^7^. Furthermore, CS influences the alternation in the DNA methylation profile in smoker samples compared with that in non-smoker samples, which implies that CS could induce adverse effects on cellular activities^7^. Thus, CS stimulates the development of diseases by interfering with genetic and epigenetic regulation^7^.

Long non-coding RNAs (lncRNAs) are RNA molecules that contain more than 200 nucleotides but lack protein function^8^. lncRNAs are involved in biological processes that determine the fate of cells^8^. Recently, understanding has increased concerning the role of lncRNA, particularly lymphoid enhancer-binding factor 1 antisense RNA 1 (LEF1-AS1), which is located on chromosome 4q25 and conserved at the transcriptional level, in biological pathways^9^. LEF1-AS1 (LEF1 Antisense RNA 1) is a long noncoding RNA transcribed from the antisense strand of the LEF1 (Lymphoid Enhancer Binding Factor 1) gene. The LEF1 gene encodes a transcription factor involved in Wnt/β-catenin signaling, critical for cell proliferation, differentiation, and tumorigenesis. LEF1-AS1 has been shown to modulate the expression of LEF1 by acting as a competing endogenous RNA (ceRNA) or by interacting with epigenetic modifiers^9^. Non-coding RNAs (ncRNAs), including lncRNAs, are also subject to epigenetic regulation. DNA methylation can similarly regulate lncRNA expression to protein-coding genes, affecting their function in transcriptional and post-transcriptional regulation. Aberrant DNA methylation patterns in lncRNAs have been implicated in various diseases, including cancer^10^. Methylation-induced silencing of tumor-suppressor lncRNAs or hypomethylation-driven overexpression of oncogenic lncRNAs can disrupt cellular homeostasis and promote malignancy. Overexpression of LEF1-AS1 is detected in several cancers, including lung cancer, colorectal cancer^11^, hepatocellular cancer, ovarian cancer, prostate cancer, and retinoblastoma^9^, and is linked with poor outcomes^12^. However, when LEF1-AS1 expression was absent, myeloid malignant cell line growth accelerated, and when LEF1-AS1 expression was restored, the proliferation process was inhibited, and the expressions of P21 and P27 were observed^13^. In addition, LEF1-AS1 expression has been identified in regulating key biological pathways such as Akt/mTOR/ERK Wnt/β-catenin and Hippo signaling^14^. A recent discovery demonstrated a positive association between LEF1-AS1 and LEF1 expressions and the development of colon cancer^11^. Thus, due to the aberrant expression of LEF1-AS1 in cancer development through the upregulation of oncogenes, hampering the expressions of tumor suppressor genes, and increase in chemotherapy resistance, the LEF1-AS1 gene is a possible target for cancer therapy^9^.

A few studies have shown the impact of CS on SNP^3^ and the altering effect of CS on DNA methylation, which causes gene expression abnormalities that induce tumorigenesis^15^. Hence, this study reports the investigation of the effect of CS on LEF1-AS1 DNA methylation status as well as the LEF1-AS1 expression.

## METHODS

### Study design

This study employs an experimental approach to investigate the effects of cigarette smoke on the methylation status and expression of LEF1-AS1 in relevant cell models and patient samples. The American Type Culture Collection supplied the cultured human umbilical vein endothelial cells (HUVECs) and CS. Clinical samples were from smokers and non-smokers (e.g. peripheral blood mononuclear cells). Participants were selected using a random sampling approach, ensuring that the study population was representative of the target group. Subjects were recruited under specific premises, e.g. hospital settings, voluntary participation, and clinical trial. Only those meeting the predefined eligibility criteria, such as age, smoking history, disease status, etc., were included in the study.

### Ethical approval and sample collection

To conduct our study, we were granted ethical approval by the research ethics committee of the College of Applied Medical Sciences at King Saud University in Riyadh, Saudi Arabia (reference No. CAMS 13/3536), allowing us to collect 64 blood samples from Saudi male participants (32 smokers and 32 non-smokers) at the Blood Donation Center of King Saud Medical City (Riyadh, Saudi Arabia) between September 2018 and December 2019. Each participant provided information about their age, medicines taken, family history of diseases, and smoking status. All participants signed a written consent form.

### DNA extraction and sodium bisulfite modification and methylation-specific (promoter region of LEF1-AS1) polymerase chain reaction

Lymphocyte genomic DNA samples from smokers and non-smokers were collected using the PureLink Genomic DNA Mini Kit (Invitrogen). Thereafter, 500 ng of the genomic DNA sample was subjected to sodium bisulfite conversion employing the EZ DNA Methylation-Gold Kit (Zymo Research), as previously explained^16^. The sodium bisulfite-treated DNA sample was amplified using polymerase chain reaction (PCR) with LEF1-AS1 methylation-specific PCR (MSP) primers.

The MSP primers for methylated DNA (mDNA) are:

- MSP-LEF1-AS1-F
  (TACGTACGGGGAATGTTTAGAAC) and
- MSP-LEF1-AS1-R
  (AAAAAAAACAAAAATATCACGTC).

The MSP primers for unmethylated DNA (umDNA) are:

- UMSP-LEF1-AS1-F
  (TTATGTATGGGGAATGTTTAGAATG) and
- UMSP-LEF1-AS1-R
  (AAAAAAAACAAAAATATCACATC).

The PCR setup for each sample contained 0.5 μL of each 10 μM primer, 1 μL of sodium bisulfite-treated DNA, and 10 μL of DreamTaq Green PCR Master Mix (2x; ThermoFisher). The PCR conditions are shown in Supplementary file Table 1. The PCR products were then visualized using 2% agarose gel with 0.5 g/mL ethidium bromide. ImageJ was then applied to define the band intensity.

### Cell lines and CS treatment

The American Type Culture Collection supplied the human umbilical vein endothelial cells (HUVECs). Cells were fed with Dulbecco’s modified Eagle medium (DMEM) (Sigma-Aldrich) having 10% fetal bovine serum and 10000 U/mL antibiotic and then kept at 37°C in a 5% CO_2_ incubator. After two passages, HUVECs were seeded in three T25 with media; each received (5x10^5^ cells). CS treatment was accomplished, according to Carithers and Moore^17^. A cigarette containing 1 mg tar yield and 0.1 mg of nicotine was placed into a smoke exposure chamber constructed of a 15 cm plastic tube connected to a submerged tube in a flask with 30 mL DMEM. Afterward, a negative pressure mechanism was applied. It was set to take 5 minutes to complete the cigarette consumption, allowing the CS to pass through the medium. The concentration was calculated in arbitrary units as 1 mL DMEM yields the whole components of one cigarette. Our stock concentration contains 30 mL DMEM mixed with 6 CS. So, 1 mL yielded 0.2 of one cigarette component. The three T25 flasks received 5 mL DMEM. Control cultures were fed CS-free DMEM (control), and 3 and 6 hours received DMEM containing CS (CS+3 and CS+6), respectively.

### RNA extraction, cDNA synthesis, and RT- PCR

The QIAzol Lysis Reagent (Qiagen) was applied to extract total RNA from the cell lines, and then 1 μg RNA was measured to synthesize cDNA using GoScript Reverse Transcriptase (Promega). The gene-specific primers of LEF1-AS1 were designed for real-time PCR (RT-PCR) assay, and the endogenous gene GAPDH was used (Supplementary file Table 2) to define the relative expression of the gene of interest. Each 20 μL reaction contained 2.5 μL of cDNA (200 ng/μL), 0.8 μL of 10 μM forward and reverse primers, and 10 μL of GoTaq qPCR Master Mix (Promega). Lastly, 5.9 μL of nuclease-free water was added, and a Prime Q real-time PCR machine was used. The RT-PCR steps were as follows: initiation of the run with 1 cycle at 95°C for 15 min and then 36 cycles at 95°C for 30 s, 58°C for 30 s, and then 72°C for 30 s, followed by 1 cycle at 95°C for 1 min, 58°C for 30 s, and 95°C for 30 s. After completing the run, the 2-ΔΔCt method defined the mRNA expression fold changes.

### Bioinformatic analysis

R2 genomic analysis and visualization platform is an online platform displaying a substantial amount of publicly available genomic data. Thus, this platform was employed to unveil the expression of LEF1-AS1 in (GSE10700); which shows the RNA expression of Normal Human Bronchial Epithelial (NHBE) cells after being exposed to whole CS (typical American brand of light cigarettes) for 15 min and left for 24 h, compared to Mock-exposed NBHE.

### Expression analysis LEF1-AS1 gene

The expression analysis of LEF1-AS1 gene was performed using the GTEx (Genotype-Tissue Expression) database^18^, which contains substantial data on gene expression across many tissue types in both male and female samples. The TPM (Transcripts per Million) metric was used to quantify expression levels. To visualize log-transformed values, log_10_ (TPM + 1), were plotted on the y-axis to standardize the data and handle values throughout a wide range.

### Expression in normal versus tumor tissues using GENT2 tool

The GENT2 tool^19^ was used to assess the expression of LEF1-AS1 in normal and malignant tissues. The study compared the expression levels of LEF1-AS1 in various cancer types (e.g. lung cancer and brain cancer) to their corresponding normal tissues. Box plots were used to visualize log_2_-transformed expression values. The y-axis of the graphs reflected gene expression levels in different tissue types. LEF1-AS1 expression was consistently higher in some cancer types (e.g. lung and brain cancer) than in normal tissues. The variation in tumor expression levels indicated tissue- and cancer-specific overexpression patterns.

### Meta-survival analysis

A meta-survival study was carried out using the Kaplan Meier plotter on numerous available datasets to determine the relationship between LEF1-AS1 expression and survival outcome. We systematically searched The Cancer Genome Atlas Program (TCGA) database using specific keywords and filters to identify relevant survival studies on LEF1-AS1 and cancer prognosis. Inclusion and exclusion criteria were applied to ensure the selection of high-quality studies. All studies presented key characteristics such as cancer-type survival outcomes analyzed. The hazard ratios (HRs) and confidence intervals (CIs) were extracted directly from studies or estimated from Kaplan-Meier survival curves using appropriate statistical tools. Several studies, including GSE31546, GSE31548, GSE19188, and others, were used to evaluate the survival impact of LEF1-AS1. Hazard ratios (HR) and 95% confidence intervals (CI) were generated for each study to determine the relationship between LEF1-AS1 expression and survival outcomes. HR ≥1 indicates higher risk (e.g. poor survival), while HR <1 suggests a protective effect. The results from many investigations were combined using both fixed-effect and random-effects models. The I^2^ statistic was used to measure heterogeneity between trials^20^.

### Functional enrichment analysis

The functional enrichment analysis was conducted using the Gene Ontology: Cellular Component gene sets pathway analysis in WebGestalt^21^ to identify gene ontology terms enriched in LEF1-AS1-related gene sets. The analysis highlighted significant enrichment of biological processes and molecular functions, such as protein serine/threonine kinase activity (GO:0004674) and regulation of TORC1 signaling (GO:1903432), with adjusted p-values indicating the significance of enrichment. Pathways related to TORC1 signaling, oxidative stress responses, and telomere maintenance were notably enriched, suggesting that LEF1-AS1 may play roles in critical cellular processes. Enrichment results were visualized using a dot plot, where the x-axis represented gene ontology terms, and the y-axis displayed –log_10_-transformed adjusted p-values, indicating the strength of statistical significance. We evaluated the LEF1-AS1 gene. This analysis allowed us to identify enriched biological processes, molecular functions, and cellular components associated with the differentially expressed genes, including members of the LEF1-AS1, in lung cancer tissues.

### Statistical analysis

Statistical analysis was performed using the SPSS Ver.22 software (SPSS Inc., Chicago, IL). Data are reported as mean ± standard deviation (SD). Two-tailed unpaired Student’s t-tests were applied, with statistically significance at: *p<0.05, **p<0.01, and ***p<0.001. Meta-survival analysis of the LEF1-AS1 gene was conducted with the data from a variety of studies (GSE31546, GSE31548, GSE19188, etc.) using fixed-effect and random-effects models. Each study provided its estimated effect (TE), standard error of the effect (seTE), and hazard ratio (HR) along with its 95% confidence interval (CI).

## RESULTS

### LEF1-AS1 downregulation is observed in NHBE cells exposed to CS

**Figure 1:**
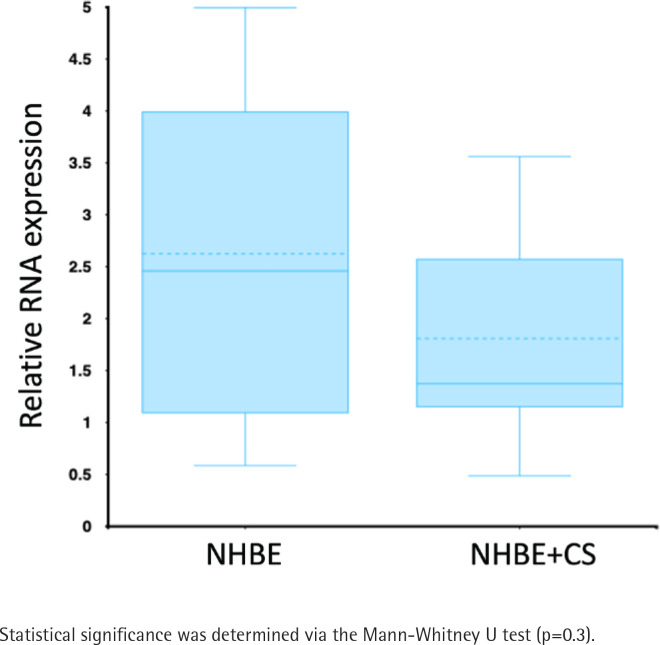
Relative RNA expression in NHBE cells with and without cigarette smoke exposure. Box plots depict median values (central line), interquartile range (IQR; box boundaries), and whiskers (1.5×IQR) of four replicate petri dish of NBEA cells. The NHBE group (control) shows higher LEF1-AS1 RNA expression compared to NHBE+CS (cigarette smoke-treated)

The expression of LEF1-AS1 in NHBE cells after being exposed to whole CS for 15 min and left for 24 h was evaluated and compared to Mock-exposed NBHE cells^22^ using R2 genomic analysis and a visualization platform. According to this study (GSE10700), LEF1-AS1 expression was reduced in CS-exposed NHBE cells compared to Mock-exposed NBHE (Figure 1).

### Clinical data of the participants

In the present study, 32 smokers and 32 non-smokers participated, and their mean ± SD ages were 31 ± 2.2 and 30.3 ± 3.4 years, respectively. Of the smokers and non-smokers, 53.1% and 40.6% were aged ≥29 years, respectively, and 46.9% of the smokers had smoked for ≥12 years, with a minimum of 10 cigarettes per day (Table 1).

**Table 1:** Characteristics of the male subjects (N=64)

*Characteristics*	*Smokers* *n (%)*	*Non-smokers* *n (%)*
**Total,** n	32	32
**Age** (years), mean ± SD	31 ± 2.2	30.3 ± 3.4
**Age** (years)		
≤30	17 (53.1)	13 (40.6)
>30	15 (46.9)	19 (59.4)
**Years of smoking**		
≥12	15 (46.9)	
<12	17 (53.1)	

### LEF1-AS1 methylation in the non-smokers and smokers

To investigate the impact of CS on the DNA methylation level of LEF1-AS1, MSP, and UMSP primers were designed at the promoter region (Supplementary file Figure 1), and 64 specimens were collected from healthy Saudi adults, of whom 32 were non-smokers, and 32 were smokers. After we extracted DNA samples and treated them with sodium bisulfite conversion, LEF1-AS1 was amplified and separated on a 2% agarose gel (Supplementary file Figures 2 and 3). The band intensities of mDNA and umDNA were then defined using ImageJ^23^. Afterward, the equation (mDNA/(mDNA+umDNA)x100 was applied to reveal the percentage of mDNA. The mean mDNA percentage for the non-smokers was 18.8 ± 5% (range: 1%–35%; Supplementary file Table 3). The samples from the smokers showed a rapid increase in DNA methylation levels. The mean mDNA percentage was 38.6 ± 3.8% (range: 22%–58%; Supplementary file Table 4).

**Figure 2:**
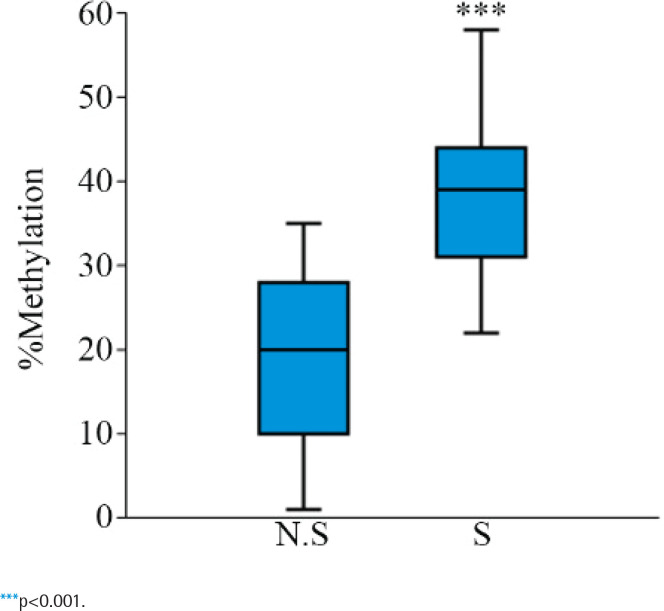
Differences in the global DNA methylation pattern of LEF1-AS1 between non-smokers and smokers. The results of the MSP analysis of LEF1-AS1 in 64 samples from non-smokers (N.S) and smokers (S) are displayed as a box plot. Each box plot indicates the mean methylation level of the 32 samples from the N.S and 32 samples from the S

Thus, our data identify that the DNA methylation level at the LEF1-AS1 promoter region significantly increased in smokers compared with non-smokers (Figure 2), which implies the impact of CS on the DNA methylation level.

### CS impedes the LEF1-AS1 expression

**Figure 3:**
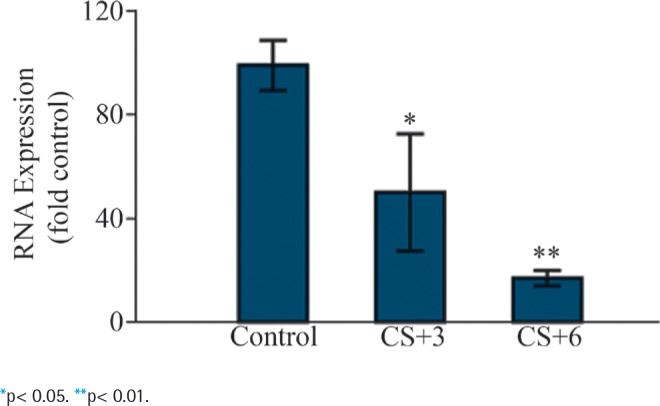
Effects of CS on LEF1-AS1 expression in HUVECs. RNA expression of LEF1-AS1 in the HUVECS after incubation with CS-free DMEM (control) and CS for 3 (CS+3) and 6 (CS+6) hours. Mean ± SD of the results from three independent experiments

To investigate the influence of CS on LEF1-AS1 expression, HUVECs were fed with media containing CS for 3 (SC+3) and 6 (SC+6) hours. We then investigated the RNA expression of LEF1-AS1, which was significantly downregulated by 45% and 83% in the SC+3 and 6 SC+6 exposed cells (Figure 3), respectively, compared with the control cells. This suggests that CS possibly contributes to the regulation of LEF1-AS1 expression.

### Tissue expression for LEF1-AS1 gene

The results indicate that LEF1-AS1 is commonly underexpressed in most tissues, with only a slight up-regulation observed in specific tissues, including the thyroid, lungs, spleen, and certain brain regions. Multiple tissue types were chosen to investigate expression patterns, including the thyroid, lung, spleen, and various brain regions. The study compared expression levels in male and female samples to see if there were any significant sex differences in specific areas like the thyroid and testis. Specifically, in tissues such as the thyroid and testis, where there may be changes in gene expression between the sexes, the expression patterns reveal modest distinctions between the sexes (Supplementary file Figure 4A). The consistent low levels observed across different tissues raise the possibility that LEF1-AS1 is not a gene that is expressed everywhere but has functions that are particular to certain tissues. The gene may contribute to localized cellular functions, and the expression differences could indicate tissue-specific regulatory mechanisms, its panel’s heatmap also displays the tissue-specific expression levels of several LEF1-AS1 isoforms (Supplementary file Figure 4B). The color gradient depicts TPM readings with purple representing low expression and yellow showing high expression. This subfigure depicts the hierarchical clustering of LEF1-AS1 transcript isoform expression across different tissues. The expression levels are represented using TPM values, with a gradient color scale from light to dark purple indicating increasing expression levels. This panel highlights the fact that LEF1-AS1 expression is very low or non-existent in most tissues. Thyroid, lung, and certain brain regions exhibit moderate expression of certain isoforms (Supplementary file Figure 4C). This subfigure illustrates the expression levels of different LEF1-AS1 transcript isoforms specifically in lung tissue. The box plots display the range of TPM values for various isoforms, with each isoform represented by a unique identifier. The figure provides a detailed breakdown of transcript diversity and expression patterns within the lung.

### Transcript expression of LEF1-AS1 in lung

The expression levels of various LEF1-AS1 transcripts (isoforms) in lung tissue are shown using the box plot. This tissue’s expression levels (TPM) vary, with each box corresponding to a distinct isoform. Certain transcripts, like ENST00000512637.5, exhibit higher expression levels than others, which are markedly lower. Only a small part of the LEF1-AS1 transcripts may be functionally significant in lung tissue based on the differential expression among isoforms (Supplementary file Figure 4). Differential transcript expression may indicate isoform-specific activity in lung tissue; certain transcripts may be involved in immune response, development, or tissue homeostasis, among other lung-specific functions. Conversely, low expression levels of some isoforms may indicate that the lung does not require them or that they are only weakly active.

### Expression of LEF1-AS1 gene across the normal and tumor tissues

**Figure 4:**
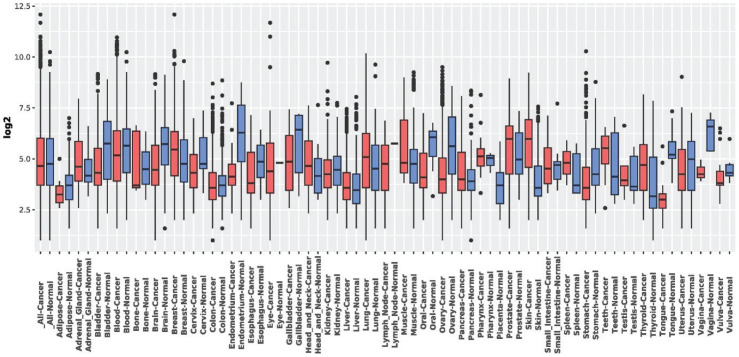
Comparative tissue-wide expression profile of LEF1-AS1 across different cancer types (represented by ‘Cancer’) and their corresponding normal tissues (represented by ‘Normal’). The data are shown in box plots, where the y-axis represents the log_2_-transformed expression levels of LEF1-AS1 in various tissue types

The expression of LEF1-AS1 gene across the normal and tumor tissues was analyzed in GENT2 tool. There is significant variation in the expression levels of LEF1-AS1 between different cancer types and their corresponding normal tissues. Some cancer types exhibit consistently higher expression levels (e.g. lung cancer, brain cancer), while others exhibit more mixed results or lower overall expression levels (Figure 4). The expression levels of LEF1-AS1 are higher in certain cancer types, such as adrenal gland-cancer, skin-cancer, and bone cancer, than in their normal counterparts. This may suggest that this gene exhibits an overexpression pattern specific to malignancy. The expression levels of LEF1-AS1 in normal tissues are also subject to variation. For example, the expression levels of normal adipose, blood and tongue tissues appear to be higher than those of the corresponding malignancies, whereas other normal tissues, such as the pancreas, exhibit levels that are more like those of their cancerous counterparts. The broad spectrum of whiskers and outliers in most box plots implies a significant degree of variability from one sample to another, which implies the possibility of heterogeneity in LEF1-AS1 expression within individual malignancies and normal tissues (Figure 4).

### Meta-survival analysis of LEF1-AS1 gene

This analysis was conducted using data from various studies (GSE31546, GSE31548, GSE19188, etc.) using fixed-effect and random-effects models. Each study provided its estimated effect (TE), standard error of the effect (seTE), and hazard ratio (HR) along with its 95% confidence interval (CI) (Supplementary file Figure 5). The hazard ratios (HR) exhibit a broad range, from near zero, suggesting no significant association, to larger values such as 12.02 for GSE31546. However, the wide confidence interval (CI), which includes 1, indicates a high degree of uncertainty and a lack of statistical significance in some cases. Most individual studies are grouped around HR=1, with a few suggesting modestly elevated risks (e.g. GSE31546, GSE19188). However, the wide confidence intervals suggest that the results are variable. The I^2^ (heterogeneity) value of 16% indicates that the included studies had low heterogeneity, indicating that the outcomes of the studies are comparatively consistent (Supplementary file Figure 5). There is also no significant heterogeneity, as indicated by p= 0.26.

### Functional enrichment analysis

Gene enrichment analysis was performed using G:Profiler, a publicly available web service. Protein annotations were made using the Gene Ontology (GO) database of biological processes. Stratified enrichment studies were conducted using disease-specific LEF1-AS1 as a query. The results show that protein serine kinase activity (GO:0106310) and protein serine/threonine kinase activity (GO:0004674) are among the significantly enriched molecular functions, with adjusted p-values of 2.658×10^-2^ and 4.650×10^-2^, respectively (Supplementary file Figure 6). These terms are involved in phosphorylation, which is critical in regulating cellular processes. Heterocyclic compound binding (GO:1901363) and Methylmalonyl-CoA mutase activity (GO:0004494) also show enrichment, indicating that genes related to these molecular binding and metabolic activities are overrepresented in the query. Notably enriched biological processes include regulation of TORC1 signaling (GO:1903432), with an adjusted p=2.800×10^-3^. TORC1 (Target of Rapamycin Complex 1) signaling is a critical cellular growth, metabolism, and survival pathway. Cellular response to reactive oxygen species (GO:0034614) and protein autophosphorylation (GO:0046777) also appear enriched, suggesting involvement in oxidative stress responses and post-translational modifications, respectively (Supplementary file Figure 6).

## DISCUSSION

This study was conducted to evaluate the role of CS in regulating LEF1-AS1 methylation levels and the impact of CS on LEF1-AS1 expression. Our data show that among adult Saudi men, LEF1-AS1, an essential regulator of cancer development, is significantly hypermethylated in smokers, increasing the mean DNA methylation rate by 19.8% compared with that in non-smokers. This implies that CS controls the epigenetic status of LEF1-AS1 by partially regulating DNA methylation. Also, exposing HUVECs to CS caused an inhibition of LEF1-AS1 expression, suggesting the possible involvement of CS in regulating the expression of LEF1-AS1.

Recent studies have investigated the influence of CS on cell fate. Published data have shown that carcinogenic compounds in CS^1^ caused disruptions of genetic and epigenetic profiles, leading to the development of some diseases^24^, including respiratory syndromes, cardiovascular diseases, and cancers. CS is the main environmental factor in altering DNA methylation status, which interrupts several biological pathways such as apoptosis, proliferation, and cell activity^25^. The GENT2 tool was used to analyze the expression of LEF1-AS1 across normal and tumor tissues, and the results revealed significant variance in expression between cancer types and their corresponding normal tissues. LEF1-AS1 expression was significantly higher in malignancies than normal tissues, indicating a possible role in carcinogenesis. Other malignancies, such as adipose, blood, and tongue tumors, showed a lower pattern of elevated expression, showing that LEF1-AS1 may be overexpressed in certain cancer types. Prior large-scale DNA methylation studies have established that CS exposure leads to genome-wide hypomethylation, particularly at regulatory elements of tumor suppressor genes, with selective hypermethylation at CpG islands in genes associated with cancer progression^7^. However, these studies primarily focused on protein-coding genes or broad epigenetic trends. The role of lncRNAs, particularly LEF1-AS1, has remained unexplored. Previous studies analyzed genome-wide transcriptional responses to CS but did not explore integrated DNA methylation and gene expression changes at specific regulatory regions^26^. Our study uniquely highlights the methylation-mediated repression of LEF1-AS1 and its downstream functional impact. However, in some normal tissues, such as the pancreas, LEF1-AS1 expression levels were comparable to those in cancerous tissues, demonstrating that the gene’s expression does not always distinguish between normal and malignant states. Numerous studies have demonstrated the widespread epigenetic impact of cigarette smoke on various genes, notably through DNA methylation changes. For instance, studies by Zeilinger et al.^27^ and Joehanes et al.^28^ revealed that cigarette smoke causes significant changes in methylation patterns across the genome, affecting genes involved in inflammation, immune response, and cancer. However, the current study delves into a more specific mechanism by identifying LEF1-AS1 as a key target cigarette smoke effect. While previous research has often focused on protein-coding genes such as AHRR, F2RL3, and GPR15^25^, this research adds depth by showing that cigarette smoke-induced methylation also affects non-coding RNA like LEF1-AS1, which has distinct regulatory roles in gene expression. Human blood samples have been utilized to define the impact of CS on DNA methylation levels to discover new genes involved in cancer development^29^. Thus, our results show that CS increased the methylation level in the DNA samples from smokers by 19.8%, consistent with a report that showed the possible role of CS in aberrant DNA methylation in genes related to diseases and cancers^28^. The mechanism of this action could be the damage to the DNA strand by cigarette chemical components, including arsenic, nitrosamine, and formaldehyde^30^. This damage stimulates DNA repair genes and DNMT1 to protect cells from mutagenic incidents^30^. DNMT1 has been detected overexpressed in lung tissue samples from smokers compared with non-smokers^31^, which consequently changes the DNA methylation status^7^. MSP is based on bisulfite conversion, which does not distinguish between 5-methylcytosine (5-mC) and 5-hydroxymethylcytosine (5-hmC). Since 5-hmC is an intermediate in DNA demethylation and has potential regulatory roles, its presence can lead to overestimating methylation levels. This is particularly relevant in tissues with high 5-hmC abundance, such as the brain and certain cancer types, where the biological effects of 5-hmC differ from those of 5-mC^32^. MSP relies on primers targeting specific CpG-rich regions, meaning only preselected sites are analyzed. In contrast, other potentially relevant CpG sites within the gene or regulatory region remain undetected. This limited coverage may fail to capture the overall methylation landscape of a locus, missing important epigenetic modifications that contribute to gene regulation. Previous studies have established that DNA methylation is a key regulator of lncRNA expression. For instance, Bhan et al.^10^ showed that lncRNAs are subject to methylation-based silencing, similar to protein-coding genes. The research on LEF1-AS1 builds on this concept by showing how cigarette smoke can induce hypermethylation of the LEF1-AS1 promoter region, leading to its silencing. Comparatively, studies such as that of Wang et al.^33^ have demonstrated that cigarette smoke-induced methylation of lncRNA MEG3 contributes to lung carcinogenesis, reinforcing the concept that lncRNAs serve as critical epigenetic targets in smokers. In addition, other mechanisms have been revealed to indicate the role of CS in inducing hypoxia and activating a crucial enzyme for synthesizing the major element of the DNA methylation process, S-adenosylmethionine^34^. Herein, CS is an important regulating factor of the DNA methylation level of LEF1-AS1.

The link between cigarette smoke, DNA methylation, and carcinogenesis has been extensively studied, particularly in the context of lung cancer. Research by Siliva et al.^7^ demonstrated that hypermethylation of tumor suppressor genes is a key mechanism by which cigarette smoke promotes tumorigenesis. The present study suggests a similar mechanism but via a non-coding RNA. Given LEF1’s known involvement in Wnt signaling and its role in various cancers, the methylation-induced silencing of LEF1-AS1 could represent a novel pathway through which cigarette smoke promotes carcinogenesis. This finding is consistent with recent studies indicating that environmental factors, including cigarette smoke, can affect non-coding RNA function and thereby influence cancer risk^35^. High LEF1-AS1 expression levels are involved in tumorigenesis, and reducing its expression induces cell death^14^. Inversely, LEF1-AS1 expression is absent in patients with myeloid malignancies, and inducing LEF1-AS1 expression impedes the growth of AML cells^13^. Thus, LEF1-AS1 expression may be used for cancer therapy. Previous data have demonstrated the impact of CS in changing epigenetic events in important cellular pathway regulators, including lncRNA, and the possible contribution of CS to the regulation of these pathways^31^. To better understand the impact of CS on LEF1-AS1 expression, we treated HUVECs with CS due to the presence of LEF1-AS1^36^. Our findings indicate a significant reduction of LEF1-AS1 expression in the cells treated with CS in 3 and 6 hours compared with the untreated cells, which implies a possible involvement of CS in regulating LEF1-AS1. According to published data, CS stimulates the expression of DNMT1 and induces DNA methylation events^30^, as it was reported that silencing the DNMT1 expression leads to the reactivation of genes hindered by CS^31^. Therefore, CS disrupts LEF1-AS1 DNA methylation, resulting in the dysregulation of LEF1-AS1 expression. A targeted examination of LEF1-AS1 transcript expression in lung tissue revealed variable expression across multiple isoforms. The expression levels of some transcripts, including ENST00000512637.5, were higher than those of others, suggesting that only a subset of LEF1-AS1 transcripts may be functionally significant in the lungs. While low expression of other isoforms may indicate their limited or context-dependent activity in lung tissue, differential isoform expression suggests that specific LEF1-AS1 transcripts may contribute to lung-specific processes, such as immune responses or maintaining tissue homeostasis.

Altogether, this is the first reported role of CS in regulating the DNA methylation of the promoter region of LEF1-AS1 in the Saudi adult population. We also presented that LEF1-AS1 expression was downregulated after CS treatment, which implies that CS could be involved in regulating LEF1-AS1 expression through altering DNA methylation status.

### Limitations

Although the current study has shown for the first time the impact of CS on DNA methylation level and the RNA expression of LEF1-AS1, there are some limitations, including the size of study samples, few participants have agreed to enroll in the study. Also, if female samples were available, it would have strengthened the research outcomes. Besides, some biases from the enrollments might have affected the results. Also, the experiments were primarily conducted on cell lines *in vitro*, which may not fully replicate the complexity of a living organism as well as the study does not include *in vivo* experiments to confirm the findings in animal models or human subjects, which limits the ability to generalize the results to clinical settings. Additionally, the bioinformatic analysis relied on data from public databases and a relatively small number of samples from the institution, which may not represent the broader population. Finally, the findings are specific to the cell lines and conditions used in the study, and further research is needed to determine if the results apply to other cell lines, conditions, or patient populations.

## CONCLUSIONS

We demonstrated that cigarette smoke exposure modulates the methylation levels of LEF1-AS1, suppressing its expression. The analysis of LEF1-AS1 expression across tissues, its isoforms, and its association with cancer and survival outcomes reveals valuable insights into its biological roles and potential functional relevance. LEF1-AS1 is a long non-coding RNA (lncRNA) that has garnered interest due to its differential expression patterns across tissues and disease states, particularly cancers. Our findings suggest that the hypermethylation of the LEF1-AS1 promoter region may play a pivotal role in regulating the transcriptional silencing of this long non-coding RNA (lncRNA), which has previously been implicated in various cellular processes, including tumorigenesis and cell proliferation. The data provide compelling evidence that the epigenetic alterations induced by cigarette smoke may contribute to dysregulated LEF1-AS1 expression, potentially influencing downstream pathways associated with cellular differentiation, immune response, and carcinogenesis. Given the emerging role of lncRNAs in cancer biology, our results offer valuable insights into how cigarette smoke may drive malignancy through epigenetic modulation. Our findings highlight that CS significantly increases DNA methylation levels of LEF1-AS1. Also, its induction caused a significant decrease of LEF1-AS1 at the transcriptional level.

## Supplementary Material



## Data Availability

The data supporting this research are included in the article. Further information is available from the authors on reasonable request.
