# Supplementary material for: Cigarette smoke modulates methylation levels of LEF1-AS1 and impedes its expression: An experimental study
Source: Tob Induc Dis. 2025 May 2;23:10.18332/tid/203507. doi: 10.18332/tid/203507 (PMC12046985; doi:10.18332/tid/203507)
Supplement: Supplementary file 1 [file TID-23-54-s1.pdf]

Supplementary File

Tables

Table1: PCR condition used in MSP assay.

Temperature (°C)	Duration	Cycles
95	5 minutes	1
94	30 seconds	36
55	30 seconds	
72	30 seconds	
72	10 minutes	1

Table 2. Sense and antisense primers of the amplicon

**Amplicon**	**Sense Primer**	**Antisense Primer**	**Ta***
LEF1-AS1	TCTTCCGGATGCAGCCTTTT	TCAGGGTTTCTCCATTGCCC	58
GAPDH	GGGAAGCTTGTCATCAATGG	GAGATGATGACCCTTTTGGC	58

***Ta: Annealing temperature**

Table 3. Methylation level of *LEF1-AS1* in the nonsmokers

Participant number	Methylation rate (%)
**1**	23
**2**	28
**3**	19
**4**	33
**5**	14
**6**	20
**7**	10
**8**	4
**9**	10
**10**	5
**11**	3
**12**	1
**13**	26
**14**	30
**15**	28
**16**	25
**17**	25
**18**	35
**19**	32
**20**	24
**21**	14
**22**	33
**23**	29
**24**	20
**25**	19
**26**	24
**27**	14
**28**	5
**29**	11
**30**	21
**31**	5
**32**	14
**Mean methylation rate**	**18.8 ± 5**

Table 4. Methylation level of *LEF1-AS1* in the smokers

**Participant number**	**Methylation rate (%)**
**1**	45
**2**	30
**3**	42
**4**	43
**5**	31
**6**	38
**7**	49
**8**	43
**9**	37
**10**	58
**11**	44
**12**	22
**13**	38
**14**	31
**15**	25
**16**	28
**17**	32
**18**	30
**19**	43
**20**	41
**21**	31
**22**	49
**23**	39
**24**	32
**25**	43
**26**	45
**27**	43
**28**	41
**29**	45
**30**	37
**31**	37
**32**	44
**Mean methylation rate**	38.6 ± 3.8

Figures

Figure 1. Location of the *LEFA-AS1* assay. The USCS genome browser (https://genome.ucsc.edu) displays the location of the amplicon on the *LEF1-AS1* promoter and the content of the CpG island on the promoter region of *LEF1-AS1*.

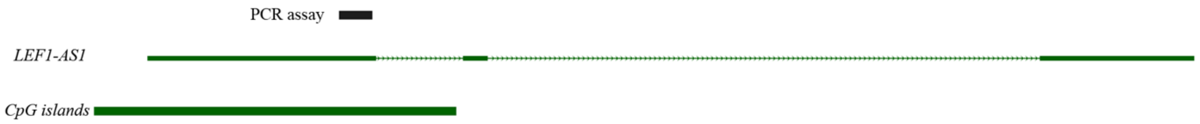

Figure 2. Methylation status of *LEF1-AS1* in the samples from the nonsmokers. The methylation level of *LEF1-AS1* was measured using MSP in 32 DNA samples extracted from nonsmokers and treated with sodium bisulfite conversion. Um and M indicate unmethylated and methylated specific primers, respectively. mDNA: fully methylated DNA; UmDNA: unmethylated DNA; N.S: nonsmoker.

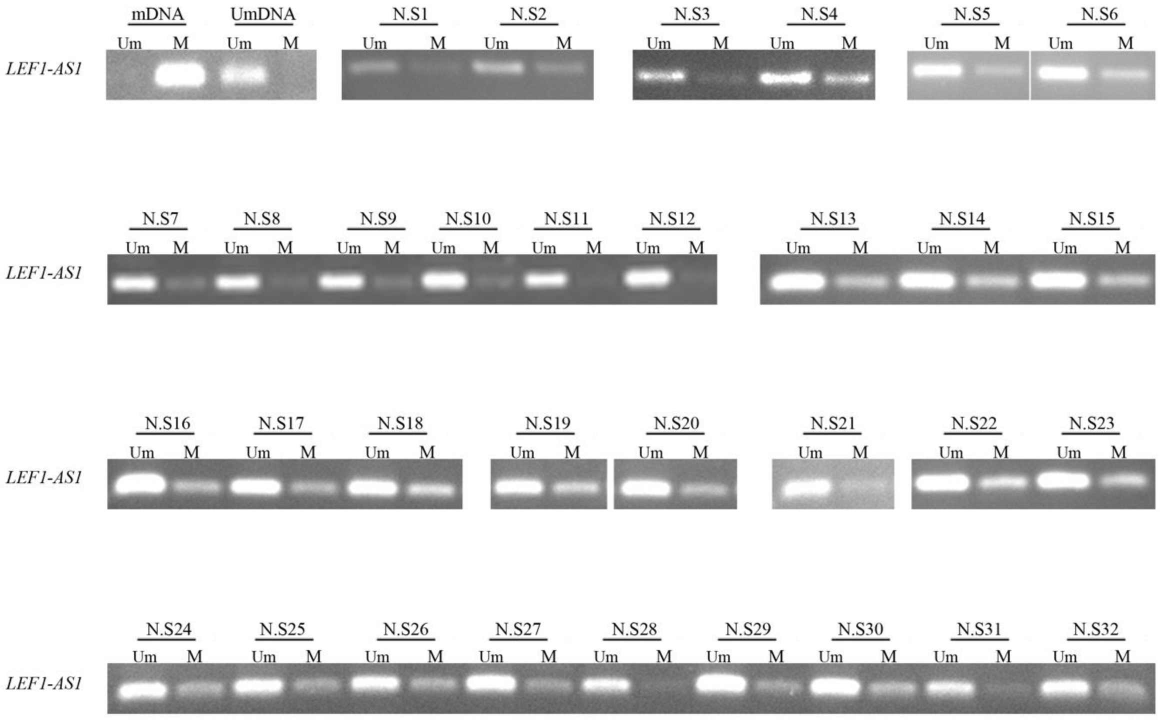

Figure 3. Methylation status of *LEF1-AS1* in the samples from the smokers. The methylation level of *LEF1-AS1* was measured using MSP in 32 DNA samples extracted from smokers (S) and treated with sodium bisulfite conversion. Um and M indicate unmethylated and methylated specific primers, respectively. mDNA: fully methylated DNA; umDNA: unmethylated DNA.

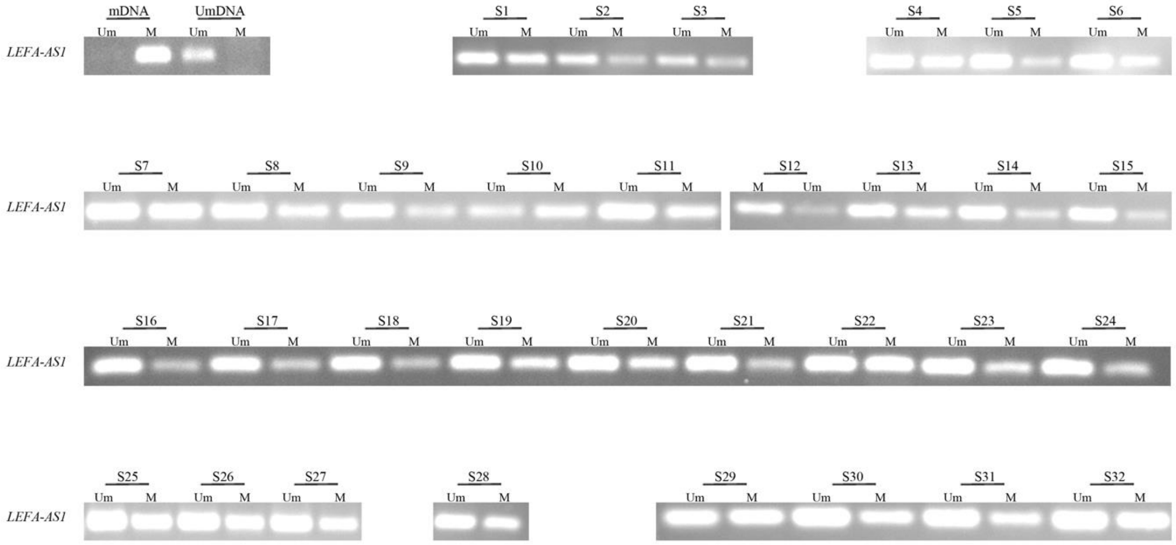

Figure 4. The expression patterns of the LEF1-AS1 gene (ENSG00000232021.6) across various tissues, highlighting its transcription levels and isoform diversity.

(A) Bulk tissue gene expression levels of LEF1-AS1 (ENSG00000232021.6) across various human tissues. Data are displayed as violin plots, with expression values represented in log10 (Transcripts Per Million, TPM+1). Male and female samples are color-coded (red for female, blue for male).

(B) Heatmap and hierarchical clustering of LEF1-AS1 isoform expression across human tissues. TPM values are represented by the intensity of the purple gradient. This subfigure depicts with a gradient color scale from light to dark purple indicating increasing expression levels.

(C) Transcript structure and expression levels of LEF1-AS1 isoforms in lung tissue. Each bar represents a transcript, with exon structures displayed as rectangles and introns as connecting lines. The expression levels (TPM) are indicated by the circle size next to each transcript.

A

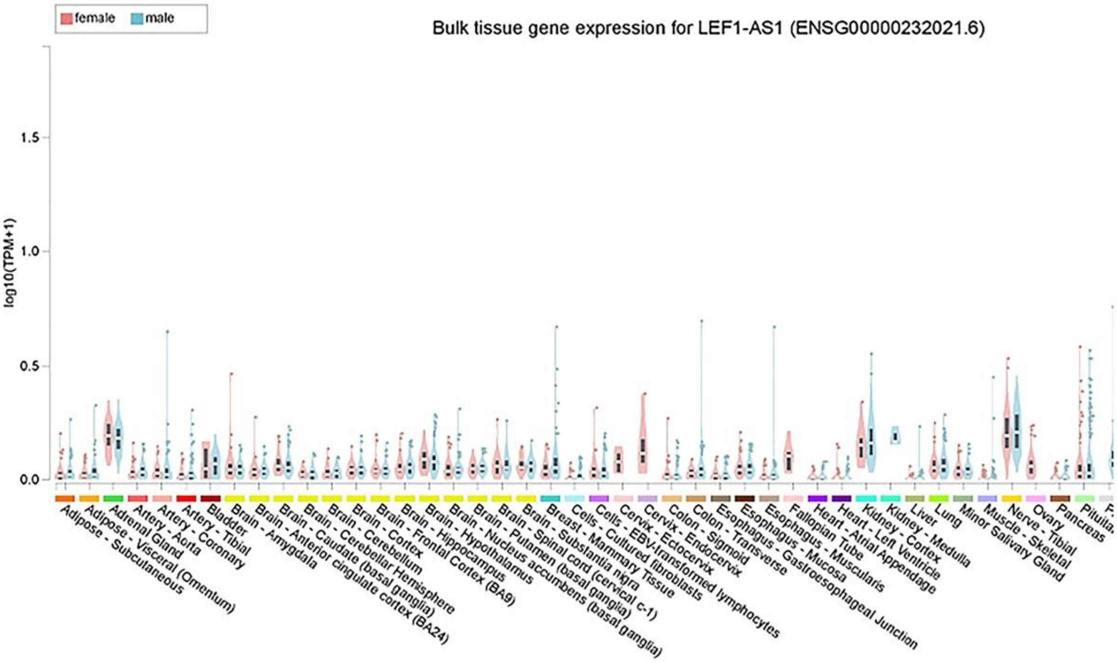
female
male
Bulk tissue gene expression for LEF1-AS1 (ENSG00000232021.6)
log10(TPM+1)
1.5
1.0
0.5
0.0

B

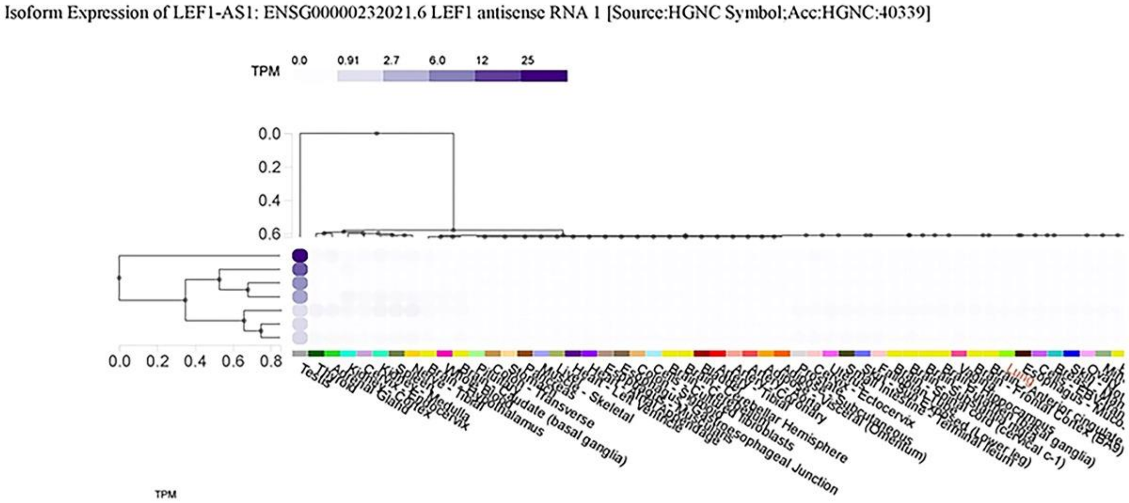
Isoform Expression of LEF1-AS1: ENSG00000232021.6 LEF1 antisense RNA 1 [Source:HGNC Symbol;Acc:HGNC:40339]
TPM
0.0
0.91
2.7
6.0
12
25

C

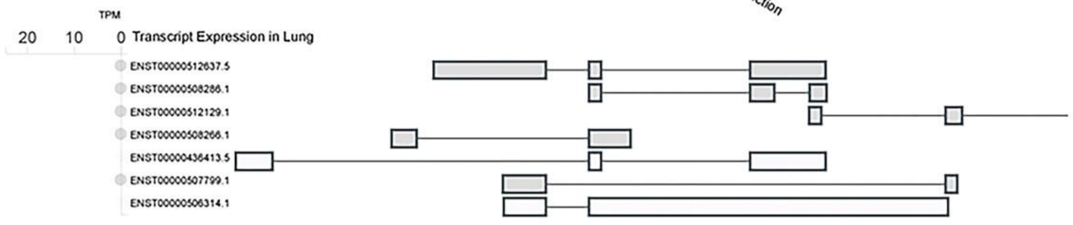
TPM
20
10
0
Transcript Expression in Lung
ENST00000512637.5
ENST00000508286.1
ENST00000512129.1
ENST00000508266.1
ENST00000436413.5
ENST00000507799.1
ENST00000506314.1

Figure 5. The forest plots depicting the results of meta-analyses that assess the association between a certain variable (possibly gene expression) and survival outcomes, presented through hazard ratios (HR). Each row represents an individual study included in the meta-analysis. The specific dataset identifiers (e.g., GSE31546-GPL570) indicate the source of the study. "TE" represents the treatment effect estimate (log HR), and "seTE" denotes the standard error of the effect estimate. The "HR" column shows the estimated hazard ratio for each study. The "95%-CI" column gives the 95% confidence interval, indicating the range within which the true effect size likely falls. Weight of Each Study (Weight Columns) indicate the contribution of each study to the overall effect size under fixed and random-effects models.
Studies with smaller standard errors (more precise estimates) receive higher weights.
Each box represents the effect estimate (HR) from an individual study.
The size of the box indicates the study's weight in the analysis.
The horizontal line through each box represents the 95% confidence interval.
The diamond at the bottom represents the overall pooled effect, with the width of the diamond reflecting the confidence interval.
The I^2 value (16% in the first plot and 50% in the second) indicates the proportion of variation due to heterogeneity rather than chance.

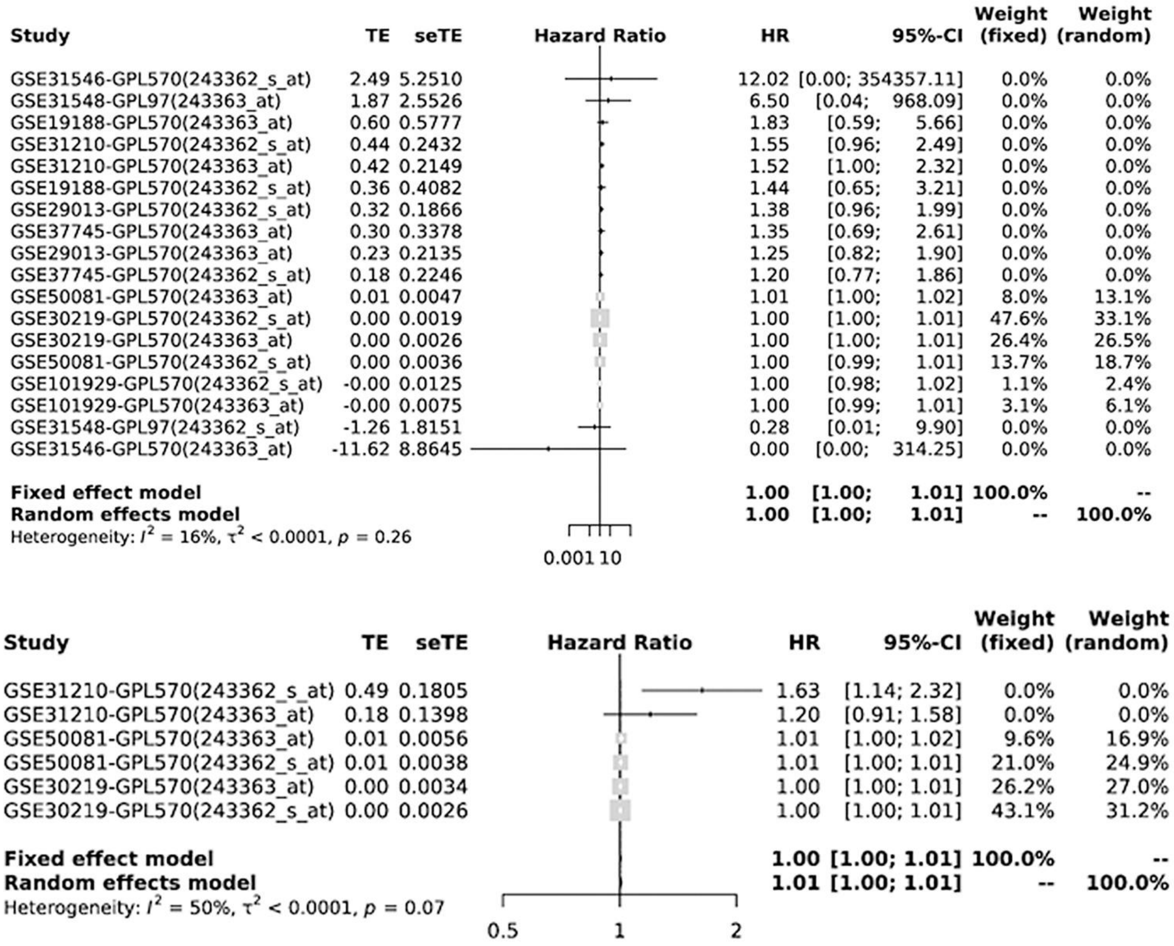

Figure 6. The enrichment is based on LEF1-AS1 gene as a query performed for the organism Homo sapiens. The x-axis categorizes gene sets into different functional terms. The colored bars at the bottom (red, orange, green, purple, etc.) represent the different gene ontology terms. The x-axis displays the functional terms that were found to be significantly influenced, with each term color-coded according to the data source. The data sources include species and type, while the sources that were ignored are shown as grey. Modified enrichment p-values on a -log10 scale are displayed on the y-axis.

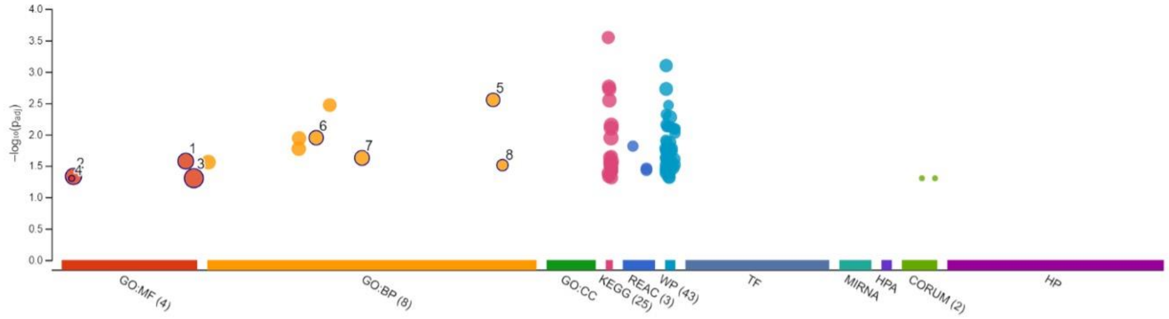

ID	Source	Term ID	Term Name	p_{adj} (query_1)
1	GO:MF	GO:0106310	protein serine kinase activity	2.658×10^{-2}
2	GO:MF	GO:0004674	protein serine/threonine kinase activity	4.650×10^{-2}
3	GO:MF	GO:1901363	heterocyclic compound binding	4.967×10^{-2}
4	GO:MF	GO:0004494	methylmalonyl-CoA mutase activity	4.990×10^{-2}
5	GO:BP	GO:1903432	regulation of TORC1 signaling	2.800×10^{-3}
6	GO:BP	GO:0034614	cellular response to reactive oxygen species	1.124×10^{-2}
7	GO:BP	GO:0046777	protein autophosphorylation	2.359×10^{-2}
8	GO:BP	GO:1904353	regulation of telomere capping	3.075×10^{-2}

**version**	e111_eg58_p18_f463989d
**date**	9/21/2024, 11:33:04 AM
**organism**	hsapiens

g:Profiler

© 2025 Almutairi B.O. et al.
